# Machine Learning-Based Investigation of Factors Influencing Recurrence of Colorectal Adenomatous Polyps: A Retrospective Cohort Study

**DOI:** 10.3390/cancers18183012

**Published:** 2026-09-17

**Authors:** Shenshen Wang, Xiaochun Zhang, Shuwen Zhang, Qing Ren, Chao Jin, Yang Yang

**Affiliations:** 1 The Second Clinical Medical College of Nanjing University of Chinese Medicine, Nanjing 210017, China; yangjinqing20@163.com (S.W.); 202430161@njucm.edu.cn (X.Z.); renqing2026@sina.com (Q.R.); 2 The First Clinical Medical College of Nanjing University of Chinese Medicine, Nanjing 210017, China; ggw146816@sina.com

**Keywords:** colorectal polyps, polyp recurrence, machine learning, dyslipidemia, predictive model

## Abstract

Objectives: Colorectal adenomatous polyps are well-recognized precancerous lesions of colorectal cancer. Patients who undergo endoscopic polypectomy still face a high risk of polyp recurrence, and individualized risk stratification remains challenging in routine clinical practice. Dyslipidemia has been implicated in adenoma development, but its role in recurrence and the predictive value of machine learning tools are understudied in Chinese populations. This study aimed to identify independent risk factors for adenoma recurrence and compare the performance of eight machine learning prediction models. Methods: This single-center retrospective cohort study included 769 patients who underwent colonoscopic polypectomy and completed at least one surveillance colonoscopy. A non-random site-based split was used to derive a training cohort (*n* = 539, Endoscopy Center) and an independent internal test cohort (*n* = 230, Colorectal Center). Univariate and multivariate Cox proportional hazards regression were applied to identify independent predictors of recurrence. Eight machine learning models were constructed using the selected predictors, and their discriminative performance, calibration, and clinical net benefit were comprehensively evaluated. Results: Abnormal high-density lipoprotein cholesterol (HDL-C), higher baseline polyp count, and larger total polyp volume were independent risk factors for adenoma recurrence. In the training set, the gradient boosting machine (GBM) achieved the highest AUC of 0.874, followed by XGBoost (AUC = 0.866); in the test set, GBM and XGBoost maintained favorable discriminative performance with AUCs of 0.863 and 0.849, respectively. The two models delivered comparable clinical net benefit across clinically relevant probability thresholds. XGBoost demonstrated acceptable calibration (Hosmer-Lemeshow *p* = 0.065), whereas GBM showed statistically significant miscalibration (*p* = 0.043). Conclusions: Abnormal HDL-C and greater baseline polyp burden are independent predictors of earlier colorectal adenoma recurrence. Machine learning models, particularly gradient boosting algorithms, achieve favorable discriminative performance and may serve as complementary tools for post-polypectomy risk stratification, though further calibration optimization is warranted prior to clinical application.

## Simple Summary

Colorectal adenomatous polyps are well-recognized precancerous lesions that can progress to colorectal cancer. After endoscopic removal, patients remain at high risk of recurrence, and tailored risk stratification is difficult to implement in routine clinical practice. This retrospective study of 769 Chinese patients identified independent predictors of adenoma recurrence and benchmarked the performance of eight machine learning prediction models. Abnormal high-density lipoprotein cholesterol, greater baseline polyp count, and larger total polyp volume were independently linked to higher recurrence risk. Gradient boosting algorithms achieved the best discriminative performance, with XGBoost 3.2.1.1 showing reliable calibration. These tools may help clinicians personalize post-polypectomy surveillance strategies and improve secondary prevention of colorectal cancer.

## 1. Introduction

Colorectal polyps, particularly adenomatous polyps, are widely recognized as the most important precancerous lesions of colorectal cancer (CRC). CRC is the third most commonly diagnosed malignancy and the second leading cause of cancer-related deaths worldwide [1]. Endoscopic polypectomy is currently one of the most effective modalities for CRC prevention. However, clinical practice indicates that patients after polypectomy have a high risk of polyp recurrence, and some may even develop advanced adenomas or CRC [2]. Therefore, identifying and intervening in risk factors for polyp recurrence is of great public health and clinical significance for developing individualized surveillance strategies and optimizing secondary prevention of CRC.

Although colonoscopy is considered the “gold standard” for colorectal polyp screening and surveillance, consensus on the optimal surveillance interval remains lacking. In clinical practice, risk stratification based on baseline polyp characteristics—such as number, size, and histological type—is necessary to individualize management [3]. Current international guidelines emphasize differentiating low-risk from high-risk lesions to tailor surveillance strategies. Retrospective evidence further indicates that baseline adenoma features significantly influence recurrence risk and appropriate surveillance timing. For example, the optimal surveillance interval for low-risk patients with non-advanced adenomas is approximately three years; in contrast, patients with advanced adenomas who have high-risk features (e.g., male sex, larger adenoma diameter, right-sided colonic distribution) require more intensive and individualized follow-up [4]. These findings reinforce the clinical concept of risk stratification based on baseline polyp characteristics to implement differentiated surveillance, thereby optimizing monitoring strategies by balancing recurrence risk and healthcare resource burden.

In recent years, the association between metabolic abnormalities and the development of gastrointestinal tumors has attracted increasing attention. High dietary fat intake is considered a risk factor for inducing premalignant lesions (e.g., adenomatous polyps) and exacerbating colorectal tumorigenesis [5]. Elevated circulating lipids create a favorable microenvironment for abnormal proliferation of colorectal epithelial cells by promoting cell proliferation, inducing oxidative stress and chronic inflammation, or affecting bile acid metabolism [5,6]. Although several studies have investigated the association between serum lipid levels and the risk of first occurrence colorectal polyps, the findings remain inconsistent [7,8]. A meta-analysis of 37 studies confirmed that dyslipidemia, characterized by elevated serum triglycerides (TG), total cholesterol (TC), low density lipoprotein cholesterol (LDL-C), and decreased high density lipoprotein cholesterol (HDL-C), as well as obesity, is associated with an increased risk of colorectal polyps [8]. However, a study involving 1799 participants showed no statistically significant association between serum lipid levels and colorectal adenoma occurrence [9]. More importantly, research on the relationship between serum lipid levels and the risk of polyp recurrence after polypectomy is relatively scarce, and the limited available evidence is also contradictory. For example, a Mendelian randomization analysis demonstrated that TC levels were significantly positively associated with the risk of colon polyps (all *p* < 0.05), suggesting that dyslipidemia may be a causal risk factor for polyp development [10]; however, other studies have failed to confirm a significant association between hyperlipidemia or LDL-C and recurrence risk, which might be secondary to changes in patients’ metabolic or nutritional status, or could be attributable to detection bias and confounding bias [11].

Given that the dietary structure, genetic background, and metabolic characteristics of the Chinese population differ from those of Western populations, and that there is a paucity of high-quality studies specifically addressing polyp recurrence in China, it is particularly necessary to conduct relevant research in this population. Therefore, we designed a retrospective cohort study to systematically analyze the factors influencing recurrence of colorectal adenomas.

## 2. Materials and Methods

### 2.1. Study Design

This study was a single-center, retrospective, observational cohort study. The study protocol was approved by the Ethics Committee of the Second Affiliated Hospital of Nanjing University of Chinese Medicine on 29 January 2026 (approval number: 2026SEZKY-008-01). All participants provided written informed consent.

To evaluate the generalizability of the models across distinct clinical settings and to avoid data leakage, we established a training set and an internal test set using a non-random, site-based split strategy. The training set included all patients who underwent colonoscopy at the Endoscopy Center of our hospital between January 2025 and February 2026 and met the eligibility criteria. The test set comprised all patients who attended the Colorectal Center of our hospital during the same period and fulfilled the same eligibility criteria.

### 2.2. Inclusion and Exclusion Criteria

Inclusion criteria: (1) age ≥ 18 years; (2) complete records of colonoscopic polypectomy and pathological reports available at our hospital; (3) at least one regular follow-up colonoscopy performed at least 6 months after the initial polypectomy, with complete documentation of follow-up findings (number, location, size, and pathological type of polyps).

Exclusion criteria: (1) previous diagnosis of inflammatory bowel disease or hereditary polyposis syndrome; (2) history of CRC or previous colorectal resection; (3) inadequate bowel preparation (Boston Bowel Preparation Scale score < 6) before baseline or follow-up colonoscopy, or failure to reach the cecum; (4) diagnosis of non-polypoid lesions (e.g., inflammation, lipoma, spindle cell tumor, schwannoma, neuroendocrine tumor, etc.).

### 2.3. Data Collection and Variable Definitions

Data were collected by two independent researchers from the hospital’s electronic medical record system and endoscopy information system. Baseline data included demographic characteristics (age, sex). Serum lipid profiles were obtained from fasting venous blood samples collected at the baseline colonoscopy (i.e., the initial examination when polyps were first detected). The main lipid parameters included TC, TG, HDL-C, and LDL-C. All laboratory tests were performed using standardized procedures in our hospital’s laboratory. Dyslipidemia was diagnosed according to the Chinese Guidelines for the Management of Dyslipidemia in Adults [12], with the following cutoffs: TC ≥ 5.2 mmol/L, or TG ≥ 1.7 mmol/L, or LDL-C ≥ 3.4 mmol/L, or HDL-C < 1.0 mmol/L (for men)/<1.3 mmol/L (for women).

Polyp characteristics and recurrence definition: Features of polyps were recorded at baseline and follow-up colonoscopies. Polyp recurrence was defined as the detection of new adenomatous polyps (confirmed by pathology) during follow-up colonoscopy. The number of adenomatous polyps, as well as the longitudinal and transverse diameters (mm) of the largest polyp, were recorded at both baseline and follow-up examinations. The volume of a single polyp was estimated using the simplified ellipsoid formula: V = 1/2 × (long diameter) × (short diameter)^2^ [13]. The cumulative volume at a given site was calculated as the sum of the volumes of all adenomatous polyps at that site (mm^3^).

### 2.4. Machine Learning Modeling

Based on the set of locked predictor variables identified above, the following machine learning models were developed and tuned using five-fold cross-validation within the training set framework:

Logistic Regression (LR): Used as the baseline model.

Elastic Net (ENET): The elastic net mixing coefficient α was fixed at 0.5 to balance L1 and L2 regularization, while the regularization penalty strength λ was optimized through cross-validation.

Random Forest (RF): Constructed with an ensemble of 1000 classification trees, with a minimum node size of 5 observations to control overfitting.

Support Vector Machine (SVM): Implemented with a radial basis function kernel to capture non-linear associations; the cost regularization parameter was tuned via cross-validation.

Gradient Boosting Machine (GBM): Specified with a Bernoulli distribution for binary outcomes; key hyperparameters including number of trees, maximum interaction depth, and learning rate (shrinkage) were optimized iteratively.

XGBoost: A binary logistic objective function was used, and early stopping was applied during cross-validation to prevent overfitting.

Feed-forward Neural Network (NN): A single hidden-layer architecture with 8 neurons (the number of neurons was determined by grid search on the training set considering candidate values of 4, 8, and 16) was employed, along with weight decay regularization; training was performed using the Adam optimizer.

Stacked Ensemble: A ridge regression meta-learner was trained using the out-of-fold predictions of the base learners (RF, SVM, GBM, XGBoost, and LR) generated on the training set during cross-validation to prevent data leakage.

The final models were trained on the entire training set with the optimal hyperparameters and evaluated only once on the reserved test set. The primary evaluation metric was the area under the receiver operating characteristic curve (AUC). As secondary performance measures, we calculated accuracy, sensitivity, specificity, positive predictive value (PPV), negative predictive value (NPV), and the Brier score to capture discriminative performance, classification characteristics, and prediction error. The optimal classification threshold for each model was determined by maximizing the Youden index on the cross-validated ROC curve derived from the training cohort. Pairwise comparisons of AUC values between models were performed using the DeLong test on the training set, and *p*-values were adjusted for multiple testing using the Holm procedure. Calibration performance was assessed on the training set via calibration plots and the Hosmer-Lemeshow goodness-of-fit test. Decision curve analysis (DCA) was applied to evaluate the clinical net benefit of each model across a spectrum of probability thresholds relevant to clinical decision-making.

### 2.5. Statistical Analysis

Statistical analyses were performed using SPSS version 26.0 and R version 4.2.1. Continuous variables were expressed as mean ± standard deviation or median (interquartile range), and comparisons between groups were made using the independent-samples t-test or Mann-Whitney U test, as appropriate. Categorical variables were presented as frequencies (percentages) and compared using the chi-square test or Fisher’s exact test. All subsequent steps of feature selection, model development, and hyperparameter tuning were performed exclusively on the training set. The training set was not accessed in any way until the final single evaluation of the locked models.

All data preprocessing procedures were derived and fitted solely using the training cohort, and the resulting transformation parameters were subsequently applied to the held-out test set without refitting. For algorithms sensitive to feature scale—including the ENET, SVM, and NN—predictors were standardized to z-scores using the mean and standard deviation calculated exclusively from the training dataset. In contrast, tree-based ensemble models were trained on the original unstandardized feature values. For all survival analyses, the time-to-event endpoint was defined as the duration from index polypectomy to the first histologically confirmed detection of recurrent adenoma. Patients who remained recurrence-free at their last available follow-up were censored at the date of their most recent surveillance colonoscopy.

Candidate variables with a *p*-value below 0.10 in univariate Cox regression analyses conducted on the training set were entered into a multivariate Cox proportional hazards model, which was fitted exclusively to the training data using backward stepwise selection guided by the Akaike Information Criterion (AIC). Adjusted hazard ratios (HR) with corresponding 95% confidence intervals (CIs) were computed for each retained predictor. Multicollinearity among covariates was evaluated using the variance inflation factor (VIF). Variables that retained statistical significance (*p* < 0.05) in the final multivariate model were selected to form the locked predictor set, which was used for all subsequent machine learning model development.

## 3. Results

### 3.1. Patient Baseline Characteristics and Polyp Recurrence Findings

**Figure 1:**
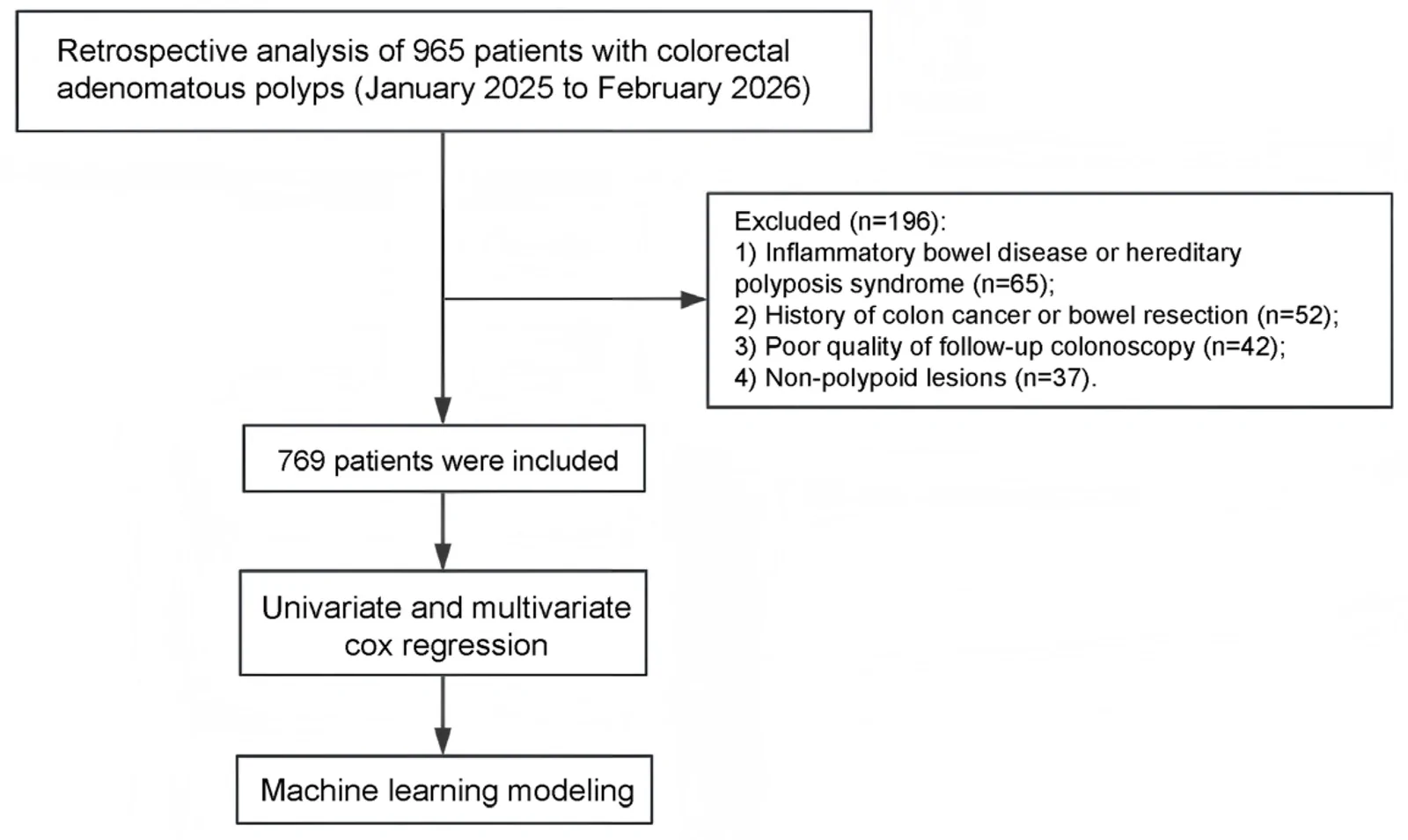
Flowchart of research design process.

A total of 769 patients with colorectal polyps were included in this study, of whom 539 comprised the training set (from the Endoscopy Center) and 230 comprised the independent test set (from the Colorectal Center). Overall, 442 patients (57.48%) experienced polyp recurrence, and 327 (42.52%) did not. The data screening process is illustrated in Figure 1. Comparisons of baseline demographic and clinicopathological characteristics between the two groups are presented in Table 1.

Intergroup comparisons showed that, except for TC, HDL-C, and the surveillance interval, there were no statistically significant differences between the recurrence and non-recurrence groups in terms of age, sex, TG, LDL-C, body mass index (BMI), or the number, volume, or maximum diameter of polyps detected at baseline (all *p* > 0.05). Specifically, the proportion of patients with abnormal TC was significantly higher in the recurrence group than in the non-recurrence group (28.73% vs. 14.98%, *p* < 0.001), while the proportion with abnormal high-density lipoprotein cholesterol was significantly lower (6.33% vs. 10.40%, *p* = 0.041). Moreover, the median surveillance interval was longer in the recurrence group (14 months vs. 12 months, *p* < 0.001).

### 3.2. Cox Regression Analysis of Factors Associated with Polyp Recurrence

Univariate Cox regression was performed for each candidate variable, including age, sex, TC, TG, HDL-C, LDL-C, BMI, and baseline polyp number, volume, and maximum diameter. As shown in Table 2, baseline polyp number (HR = 1.042, 95% CI: 1.017–1.067, *p* = 0.001), baseline polyp volume (HR = 1.0005, 95% CI: 1.0003–1.0007, *p* < 0.001), and baseline maximum polyp diameter (HR = 1.021, 95% CI: 1.008–1.035, *p* = 0.002) were significantly associated with recurrence risk. HDL-C showed borderline significance (HR = 1.375, 95% CI: 0.997–1.895, *p* = 0.052). No significant associations were observed for age, sex, other lipid parameters, or BMI (all *p* > 0.05).

Variables with *p* < 0.10 in univariate analysis were entered into a multivariate Cox model with backward stepwise selection based on the Akaike information criterion. Three independent predictors were retained: HDL-C, baseline polyp number, and baseline polyp volume (Table 2). Although significant on univariate analysis (HR = 1.021, *p* = 0.002), maximum polyp diameter was excluded from the multivariate model, due to collinearity with polyp volume and count. The overall model was significant, with a likelihood ratio χ^2^ of 29.63 (*p* = 2 × 10^−6^) and a Wald χ^2^ of 42.57 (*p* = 3 × 10^−9^). Specifically, abnormal HDL-C was associated with a 46.1% increase in recurrence risk (HR = 1.461, 95% CI: 1.059–2.017, *p* = 0.021); each additional baseline polyp conferred a 4.2% higher risk (HR = 1.042, 95% CI: 1.017–1.068, *p* < 0.001); and each 1 mm^3^ increment in baseline polyp volume corresponded to a 0.05% increase in risk (HR = 1.0005, 95% CI: 1.0003–1.0007, *p* < 0.001), underscoring polyp burden (both count and volume) as a strong predictor of recurrence.

The proportional hazards assumption was assessed using Schoenfeld residuals. The global test (χ^2^ = 4.963, df = 3, *p* = 0.174) and individual variable tests (all *p* > 0.05) indicated no violation of the proportional hazards assumption, supporting the reliability of the model estimates.

### 3.3. Performance Comparison of Machine Learning Models on the Training and Test Sets

**Figure 2:**
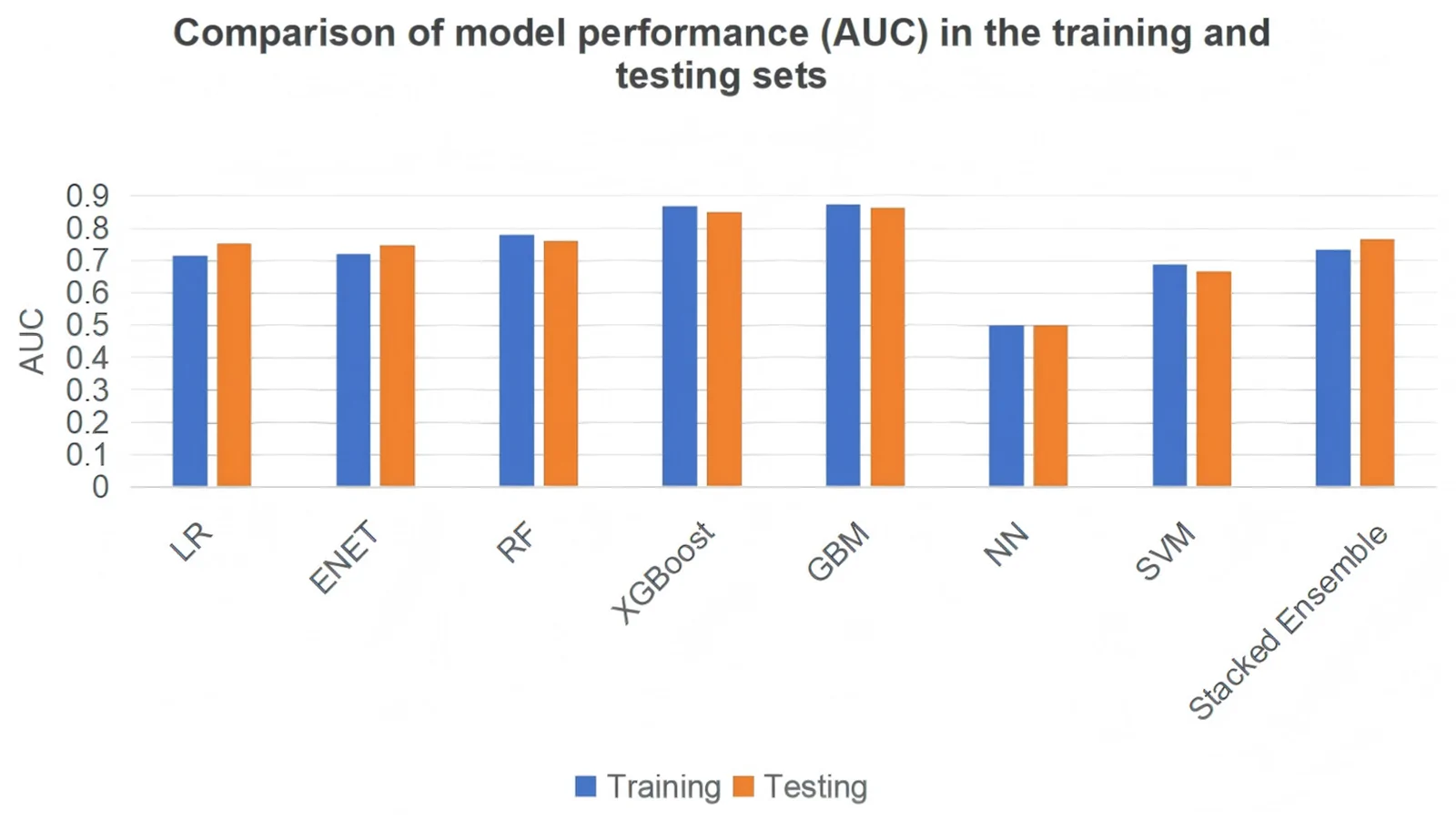
AUC comparison of eight models between the training set and the independent test set.

**Figure 3:**
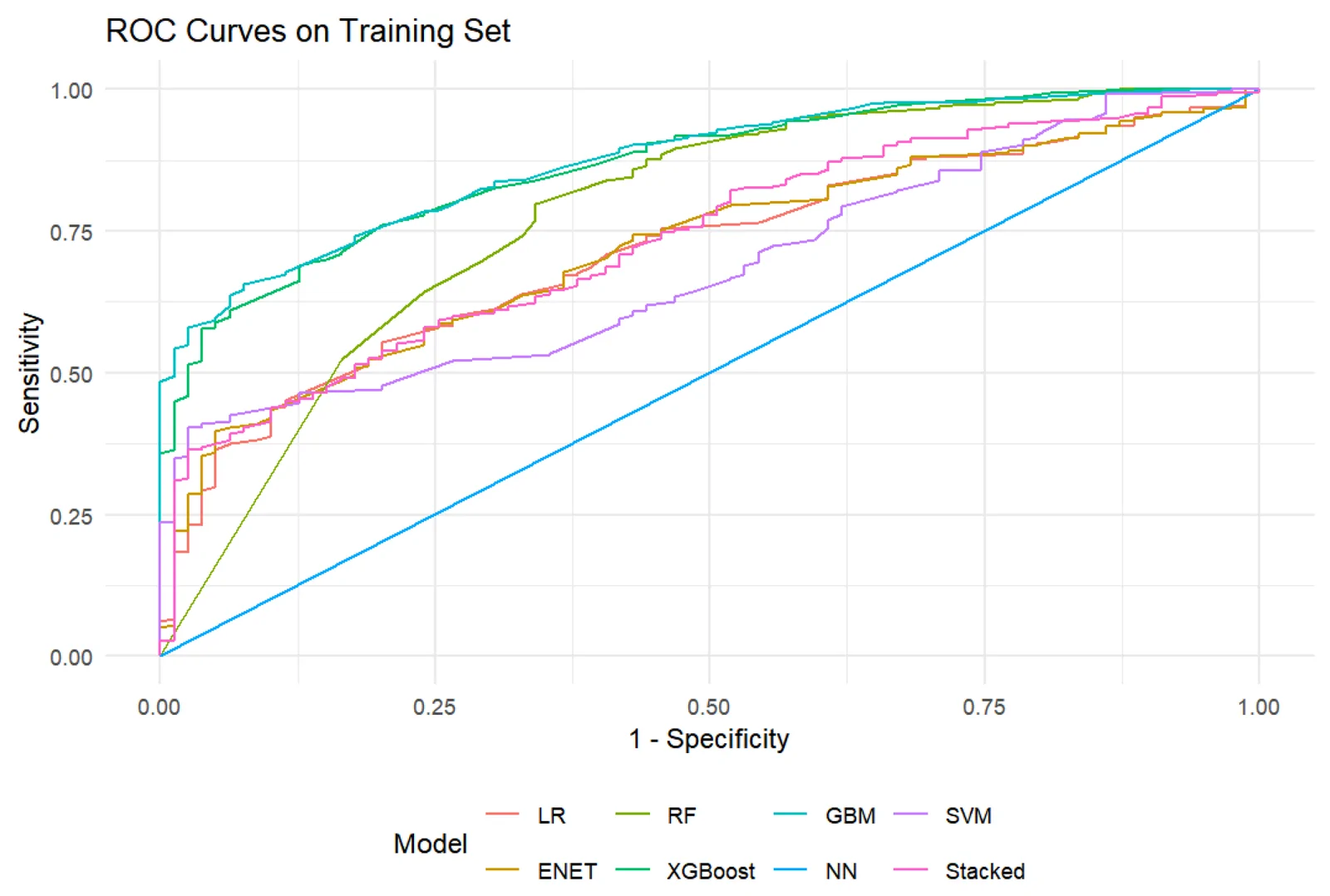
ROC curves of eight models on the training set.

Eight machine learning models—LR, ENET, RF, XGBoost, GBM, NN, SVM, and a stacked ensemble—were developed to estimate polyp recurrence risk. Model discriminative performance was assessed using the AUC and the area under the precision-recall curve (AUPRC), with 95%Cis estimated via 1000 bootstrap resamples. Results are summarized in Table 3 and Figure 2 and Figure 3.

In the training set, AUCs ranged from 0.500 (NN) to 0.874 (GBM). Tree-based ensemble models (XGBoost, GBM, and RF) achieved higher AUCs (0.778–0.874) and AUPRCs (0.938–0.976), with GBM performing best, followed by XGBoost. Linear models (LR and ENET) and SVM yielded AUCs between 0.686 and 0.719. The stacked ensemble achieved an AUC of 0.733 (95% CI: 0.680–0.785) and an AUPRC of 0.940 (95% CI: 0.916–0.959), with performance intermediate between the linear and tree-based models. In the test set, GBM maintained the highest AUC of 0.863 (95% CI: 0.798–0.922; AUPRC: 0.935), followed by XGBoost (AUC: 0.849, 95% CI: 0.783–0.910; AUPRC: 0.931). The stacked ensemble achieved a test AUC of 0.766 (95% CI: 0.701–0.826), outperforming LR, ENET, and RF, but remaining below GBM and XGBoost. RF and SVM test AUCs declined to 0.759 (95% CI: 0.679–0.835) and 0.665 (95% CI: 0.578–0.746), respectively, while NN showed no predictive capacity (AUC = 0.500). The suboptimal performance of the neural network is likely attributable in part to the modest training set size relative to model parameter count, rather than an inherent algorithmic limitation. AUPRC trends mirrored those of AUC in the test set, with GBM and XGBoost retaining the highest values (0.935 and 0.931, respectively), and NN the lowest (0.791).

**Figure 4:**
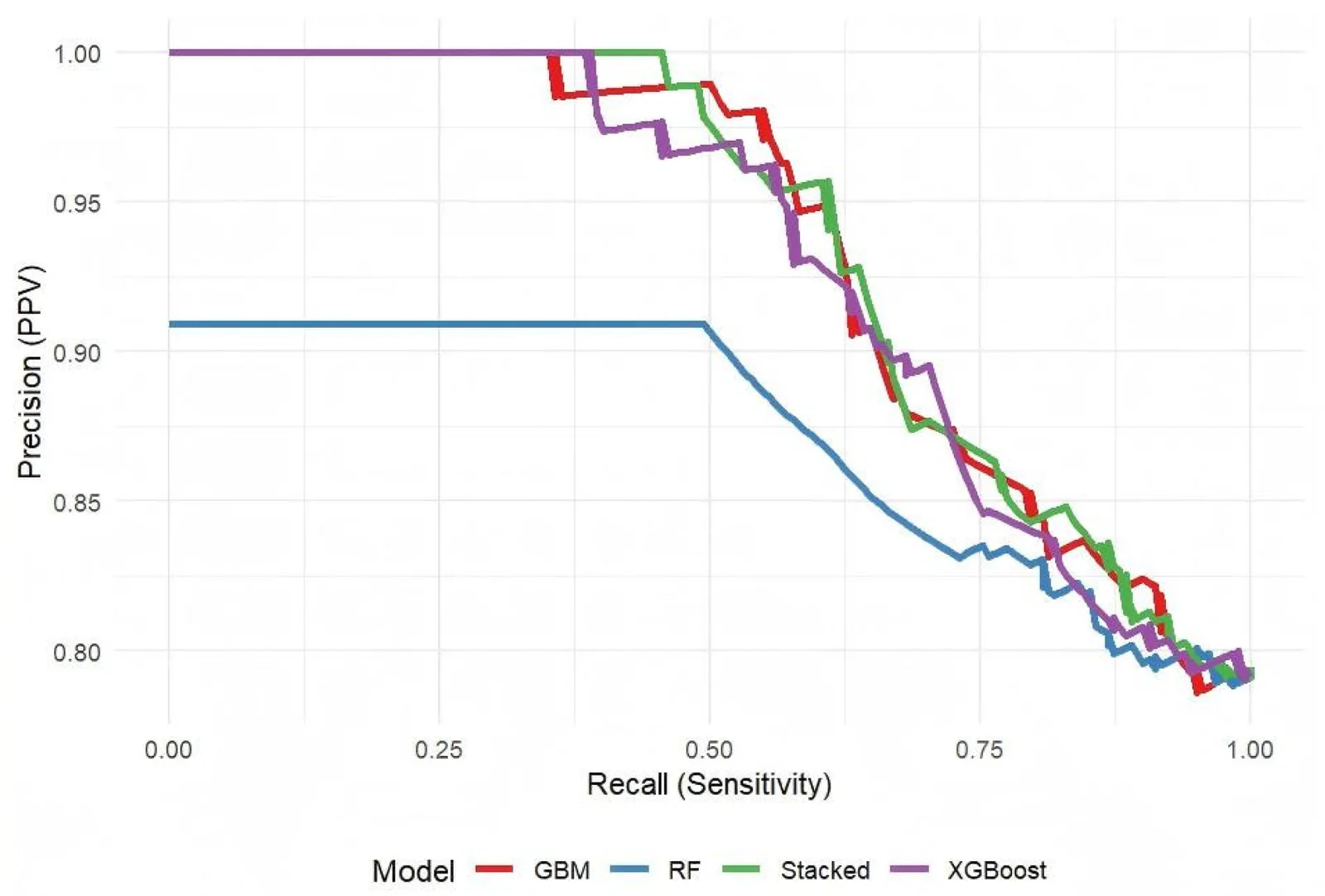
Precision-Recall curves of the top four models ranked by AUC in the training set.

To further compare the four best-performing models (GBM, XGBoost, RF, and Stacked Ensemble, ranked in descending order by training set AUC), their respective precision-recall curves were plotted (Figure 4). The PR curves for GBM, XGBoost, and the Stacked Ensemble largely overlapped across the recall range and maintained consistently high precision, indicating comparable and superior classification performance. In contrast, the RF curve fell below the others throughout, reflecting substantially lower precision at equivalent recall levels. These findings were consistent with the AUC comparisons derived from the ROC analysis, suggesting that gradient-boosting ensembles offer relatively better classification performance when the predictor space is limited.

Pairwise comparisons of the training set AUCs were performed using the DeLong test, and the resulting *p*-values were adjusted for multiple comparisons using the Holm method (Table 4). No significant difference was observed between GBM and XGBoost (raw *p* = 0.4464, adjusted *p* = 1.000). RF had a significantly lower AUC than GBM (raw *p* = 0.0097, adjusted *p* = 0.0486), but its difference from XGBoost did not reach statistical significance after adjustment (raw *p* = 0.0314, adjusted *p* = 0.1257). The Stacked Ensemble significantly outperformed RF (raw *p* = 0.0016, adjusted *p* = 0.0097), while showing no significant differences compared with GBM or XGBoost (adjusted *p* = 1.000 for both). Together, these results indicate that GBM and XGBoost achieved comparable and favorable discriminative performance.

### 3.4. Head-to-Head Comparison of Top-Performing Models and Clinical Applicability

Optimal probability thresholds for XGBoost and GBM were determined in the training set by maximizing the Youden index while constraining sensitivity to ≥ 0.80, yielding thresholds of 0.797 and 0.787, respectively. The classification performance of the two models in the training set is summarized in Table 5.

Both models demonstrated high sensitivity (XGBoost: 0.826; GBM: 0.837) and positive predictive value (0.941 for both), indicating reliable detection of recurrence. Specificity was identical at 0.696 for both models, whereas negative predictive values were modest (0.407 for XGBoost and 0.423 for GBM), suggesting limited ability to confidently rule out recurrence. Overall accuracy and F1 scores were 0.807 and 0.880 for XGBoost, and 0.816 and 0.886 for GBM, respectively.

**Figure 5:**
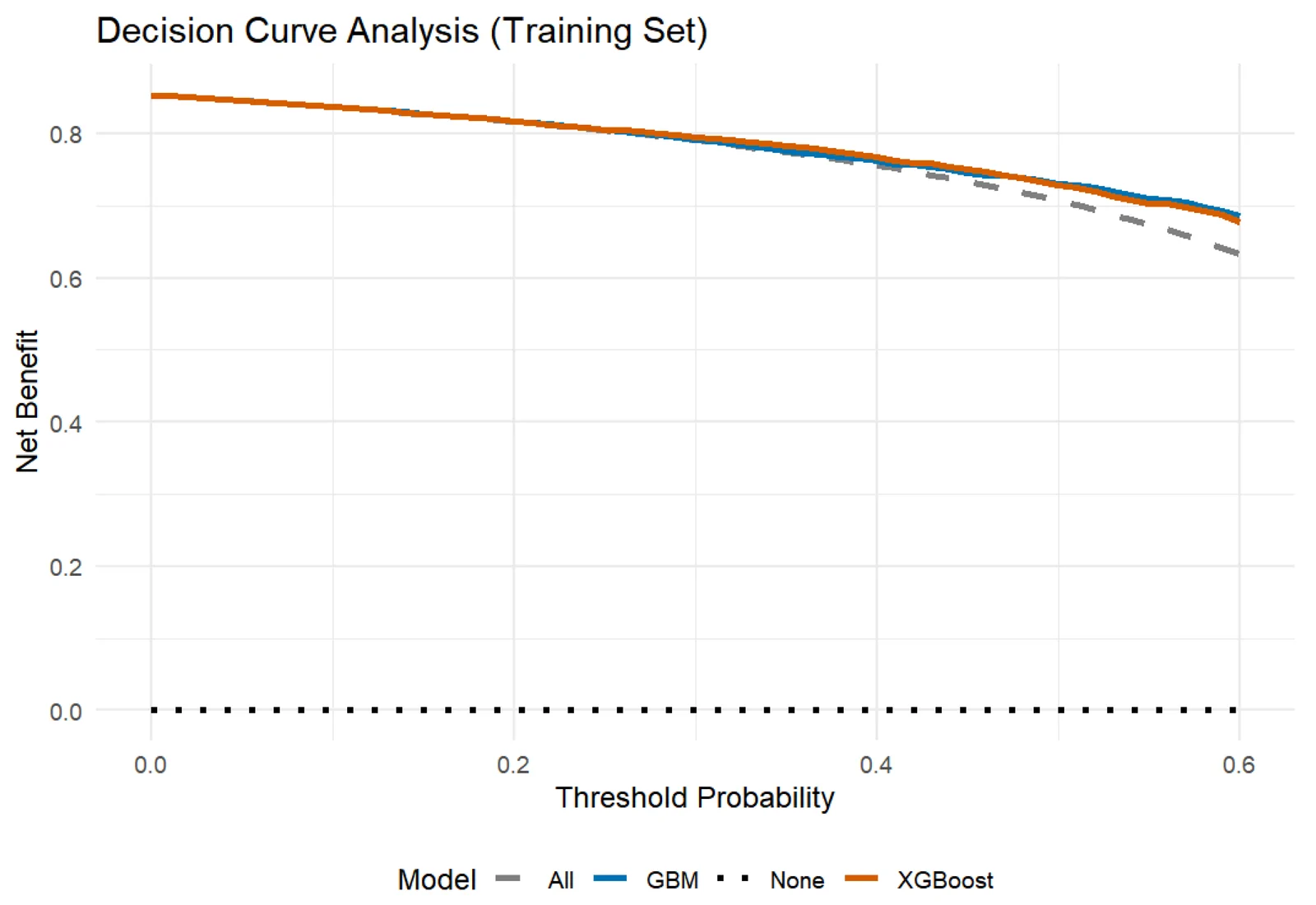
DCA of XGBoost and GBM models.

DCA further quantified the clinical net benefit of the XGBoost and GBM models across a range of threshold probabilities (Figure 5). The net benefit curves of the two models largely overlapped within the clinically relevant threshold range, indicating no substantial difference in clinical net benefit between them.

**Figure 6:**
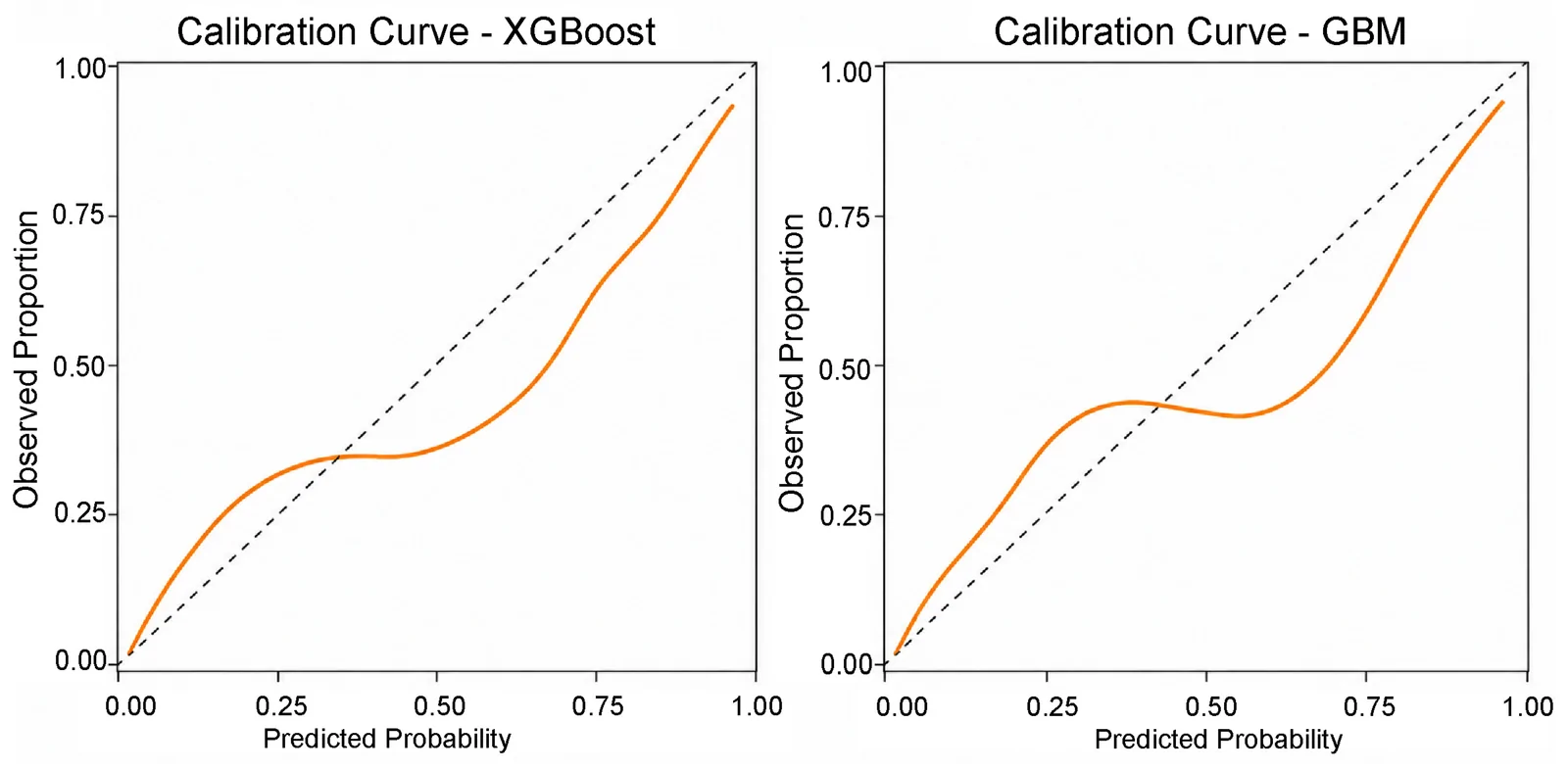
Calibration curves of the XGBoost and GBM models in the training set.

Calibration was assessed using calibration plots and the Hosmer-Lemeshow test (Figure 6). XGBoost demonstrated acceptable calibration (Hosmer-Lemeshow *p* = 0.065), whereas GBM showed significant miscalibration (Hosmer-Lemeshow *p* = 0.043). In the calibration plot, XGBoost closely followed the ideal 45° diagonal, while GBM progressively underestimated observed probabilities in the mid- to high-risk ranges. These findings suggest that probability estimates from GBM should be interpreted with caution in clinical applications.

## 4. Discussion

In this retrospective cohort study, we systematically evaluated the risk factors for recurrence of colorectal adenomatous polyps after endoscopic resection and, for the first time in a Chinese population, compared the discriminative performance of eight machine learning models for predicting polyp recurrence. The principal findings can be summarized as follows. First, elevated HDL-C, greater baseline polyp count, and larger total polyp volume were identified as independent predictors of earlier adenoma recurrence. Second, gradient boosting models (GBM and XGBoost) yielded the best discriminative performance among all algorithms, but their incremental advantage over conventional logistic regression was moderate in this low-dimensional predictor setting. Third, XGBoost demonstrated acceptable calibration, whereas GBM showed statistically significant miscalibration, highlighting the need for cautious interpretation of probability outputs from complex ensemble models in clinical practice.

### 4.1. Association Between Dyslipidemia and Polyp Recurrence

Using time-to-event analysis, we comprehensively evaluated the association between serum lipid profiles and adenoma recurrence. Abnormal HDL-C was independently associated with a 46.1% increased risk of earlier recurrence, whereas TC, TG, and LDL-C showed no significant independent associations. Consistent with our findings, Coppola et al. reported an inverse association between plasma HDL-C levels and colorectal adenoma risk after adjustment for statin use in their case-control study [14].

Potential mechanisms underlying dyslipidemia-promoted polyp recurrence have not been fully elucidated, but existing evidence suggests involvement of the following pathways. Hyperlipidemia may promote colorectal epithelial cell proliferation and inhibit apoptosis by activating the phosphatidylinositol 3-kinase/protein kinase B (PI3K/AKT) and mitogen-activated protein kinase (MAPK) signaling pathways. AKT is a serine/threonine kinase involved in multiple biological processes such as growth, proliferation, and apoptosis, and it is often overexpressed or overactivated in human CRC [15]. Furthermore, cholesterol has been shown to directly activate the PI3K/AKT pathway to promote CRC cell proliferation and survival, and mice fed a high-cholesterol diet exhibited significantly larger tumors with the highest Ki-67-positive proliferative index [16].

In addition, circulating lipid abnormalities may indirectly promote polyp recurrence and malignant transformation by affecting bile acid metabolism and gut microbiota composition [17,18]. On one hand, elevated circulating lipid levels enhance the conversion of cholesterol to bile acids, leading to an increased total bile acid pool and altered composition in the intestine. On the other hand, a high-fat diet can induce gut dysbiosis, allowing overgrowth of specific microbial populations capable of metabolizing bile acids, which in turn produce large amounts of secondary bile acids such as deoxycholic acid and lithocholic acid. Accumulation of these secondary bile acids in the gut may promote malignant transformation of colorectal epithelial cells through multiple mechanisms: (1) direct induction of oxidative DNA damage, causing base mutations and chromosomal instability [19]; (2) activation of the NF-κB inflammatory signaling pathway, promoting release of pro-inflammatory cytokines such as IL-6 and TNF-α, thereby sustaining a local chronic inflammatory microenvironment [20]; and (3) activation of the Wnt/β-catenin signaling pathway, leading to aberrant proliferation and differentiation of intestinal epithelial stem cells and accelerating adenoma formation and recurrence [21].

Moreover, long-term high-fat feeding can induce gut dysbiosis, characterized by an increased Firmicutes/Bacteroidetes ratio, a reduction in beneficial butyrate-producing bacteria (e.g., Bifidobacterium and Lactobacillus), and overgrowth of opportunistic pathogens (e.g., Escherichia and Clostridium). The resulting dysbiosis may compromise intestinal barrier function and promote adenoma formation through the following mechanisms: reduced expression of tight junction proteins, increased intestinal permeability, translocation of bacterial endotoxins such as lipopolysaccharide into the portal circulation, and induction of low-grade systemic inflammation; decreased production of short-chain fatty acids, especially butyrate, which impairs epithelial repair capacity; and activation of Toll-like receptor 4-mediated inflammatory signaling by lipopolysaccharide, further promoting colorectal epithelial cell proliferation and survival [22]. Collectively, dyslipidemia may create a positive feedback loop that promotes polyp recurrence and malignant transformation via the “gut-liver axis” bile acid metabolism pathway and the “gut-microbiota” interaction network. Nevertheless, the exact role of HDL-C in polyp recurrence remains to be elucidated by further mechanistic studies.

### 4.2. Clinical Implications of Polyp Burden and Survival Analysis

Consistent with previous literature, our study confirmed that baseline polyp burden—reflected by both polyp count and total volume—was a strong independent predictor of earlier recurrence [23]. Each additional baseline polyp was associated with a 4.2% increase in recurrence risk, and each 1 mm^3^ increment in total polyp volume conferred a 0.05% higher risk. Although maximum polyp diameter was significant in univariate analysis, it was not retained in the multivariate model due to collinearity with total polyp volume and count, indicating that composite measures of polyp burden provide more comprehensive risk stratification than single size metrics.

These findings reinforce the clinical principle of risk stratification based on baseline adenoma burden. Patients with multiple polyps or large total polyp volume face earlier recurrence and may require more intensive surveillance. From a methodological perspective, the use of Cox proportional hazards regression in this study addresses an important limitation of conventional binary outcome analysis. By modeling time to recurrence rather than simply recurrence status, the Cox approach properly accounts for differential follow-up duration across patients, which is particularly relevant for retrospective cohorts with variable surveillance intervals.

### 4.3. Performance Comparison and Clinical Applicability of Machine Learning Models

In this study, we systematically compared the performance of eight machine learning models for predicting polyp recurrence. Gradient boosting models (GBM and XGBoost) achieved the highest discriminative performance in both training and independent test sets, with training AUCs of 0.874 and 0.866 and test AUCs of 0.863 and 0.849, respectively. These models outperformed linear models (LR, ENET), random forest, support vector machine, and neural network, which is consistent with the broader literature showing that gradient boosting algorithms often deliver superior performance on tabular clinical data [24,25]. The advantage of gradient boosting models may stem from their ability to automatically capture nonlinear relationships and interactions, as well as their robustness to high-dimensional data.

At clinically optimized thresholds determined by maximizing the Youden index with a sensitivity constraint of ≥0.80, both GBM and XGBoost demonstrated high sensitivity (0.837 and 0.826, respectively) and excellent positive predictive values (both 0.941) in the training set. This performance profile makes these models well suited for identifying high-risk patients who would benefit from more intensive surveillance. However, the relatively low negative predictive values (0.423 for GBM and 0.407 for XGBoost) indicate that these models are less reliable for ruling out recurrence and de-escalating surveillance.

From a clinical practice perspective, the core predictors driving our models—polyp count and total polyp volume—align closely with the risk stratification criteria embedded in major international guidelines, including the ASGE and ESGE post-polypectomy surveillance recommendations [26,27]. These guideline-based tools typically stratify patients into broad risk tiers (e.g., low, intermediate, high) based on categorical cutoffs of polyp number and size, which guide standardized surveillance intervals. In contrast, the machine learning models developed in this study generate continuous, individualized recurrence probability estimates, which offer potential added value for personalized surveillance planning. Rather than assigning patients to fixed risk categories, these probability outputs can be used to tailor follow-up intensity on a per-patient basis, particularly for patients falling near the boundaries of conventional risk tiers. This represents a key conceptual advance over categorical risk stratification, and aligns with the broader shift toward precision medicine in gastroenterology.

### 4.4. Model Calibration and Clinical Caveats

Accurate probability calibration is critical for clinical risk communication and surveillance decision-making. In the training set, XGBoost demonstrated acceptable calibration (Hosmer-Lemeshow *p* = 0.065), with its calibration curve closely approximating the ideal diagonal line. In contrast, GBM showed statistically significant miscalibration (Hosmer-Lemeshow *p* = 0.043), characterized by systematic underestimation of recurrence probability in the mid-to-high risk range. This indicates that probability outputs from GBM should be interpreted with caution in clinical practice, as underestimation of risk in high-risk patients may lead to insufficient surveillance and delayed intervention. Post-hoc calibration methods such as Platt scaling may be applied to refine probability estimates before clinical deployment.

### 4.5. Strengths and Novelty of the Study

This study has several notable strengths. First, it adopted a retrospective cohort design and employed Cox proportional hazards regression as the primary analysis, which properly accounts for variable follow-up duration and avoids temporal bias inherent in binary outcome approaches. Second, our findings align with the landmark study by Facciorusso et al. [28], which pioneered tree-based machine learning for adenoma recurrence prognostication and identified lesion size, adenoma count, and dysplasia grade as core predictors that stratified patients into three distinct risk tiers. Building on this seminal work, our study extends the prognostic framework to a broader spectrum of colorectal adenomas, incorporates serum metabolic markers (notably HDL-C) as independent predictors, and provides the first systematic head-to-head benchmark of eight diverse machine learning algorithms against conventional regression for colorectal adenoma recurrence prediction in a Chinese population, offering empirical evidence for method selection in future clinical prediction research. Third, a site-based test set was used for validation, which minimizes data leakage and provides a more realistic assessment of model generalizability across clinical settings than random split. Fourth, model performance was evaluated from multiple dimensions including discriminative ability, calibration, and clinical net benefit, yielding a comprehensive picture of clinical utility. Finally, all included predictors are routinely available in clinical practice, conferring good feasibility for potential clinical translation.

### 4.6. Limitations of the Study

Several limitations of this study should be acknowledged. First, as a single-center retrospective cohort study, inherent selection bias and information bias may exist, and the observational design limits the ability to draw causal inferences regarding the associations between identified predictors and adenoma recurrence. Second, although the total cohort comprised 769 patients, the number of recurrence events may be insufficient for adequate training of complex machine learning algorithms such as neural networks, which likely accounts for the overfitting observed in the NN model. Third, we did not include lifestyle factors (e.g., smoking, alcohol consumption, physical activity, dietary patterns) or medication history (e.g., aspirin, statins), which may confound the relationship between serum lipid profiles and polyp recurrence. Fourth, lipid parameters were measured from a single fasting venous blood sample, which may not reliably reflect long-term systemic lipid exposure. Fifth, only internal validation with a site-based test set was conducted; external validation in independent multicenter cohorts is needed to verify the generalizability of the prediction models. Finally, the incremental discriminative value of advanced machine learning approaches over conventional regression is limited, and these findings may not extend to models built on richer data sources such as endoscopic imaging features or multi-omics biomarkers.

### 4.7. Future Directions

Future research should involve multicenter, prospective cohort studies that incorporate a more comprehensive panel of metabolic and inflammatory biomarkers (e.g., lipoprotein(a), apolipoprotein B) and adopt dynamic monitoring of lipid changes to clarify the temporal relationship between dyslipidemia and polyp recurrence. In addition, integrating deep learning models with endoscopic imaging to achieve automated extraction of polyp characteristics may further improve predictive performance. Ultimately, randomized controlled trials of clinical decision support systems are needed to validate whether machine learning-based individualized surveillance strategies can effectively improve patient outcomes and reduce healthcare costs.

## 5. Conclusions

This retrospective cohort study used Cox proportional hazards regression to systematically identify independent risk factors for postoperative recurrence of colorectal adenomatous polyps, and benchmarked the predictive performance of eight machine learning algorithms against conventional regression models. Abnormal high-density lipoprotein cholesterol, higher baseline polyp count, and larger total polyp volume were independently linked to earlier onset of adenoma recurrence. GBM and XGBoost, two representative gradient boosting algorithms, achieved favorable discriminative performance. XGBoost exhibited acceptable calibration, while GBM showed statistically significant miscalibration, calling for cautious interpretation of its probability outputs in clinical settings. Machine learning approaches may serve as complementary decision-support tools for risk stratification in post-polypectomy surveillance. Looking ahead, multicenter studies incorporating broader predictor sets are warranted to fully unlock the clinical potential of machine learning in this field.

## Figures and Tables

**Table 1:** Baseline demographic and clinical characteristics of all patients, *n* (%).

Characteristic	Overall (*N* = 769)	No Recurrence (*N* = 327)	Polyp Recurrence (*N* = 442)	*p* Value
Age, median [IQR]	60.000 [53.000, 67.000]	60.000 [52.000, 68.000]	60.000 [53.000, 67.000]	0.983
Sex				0.312
Female	269 (34.98%)	121 (37.01%)	148 (33.48%)	
Male	500 (65.02%)	206 (62.99%)	294 (66.52%)	
TC				<0.001
Normal	593 (77.11%)	278 (85.02%)	315 (71.27%)	
Abnormal	176 (22.89%)	49 (14.98%)	127 (28.73%)	
TG				0.522
Normal	548 (71.26%)	237 (72.48%)	311 (70.36%)	
Abnormal	221 (28.74%)	90 (27.52%)	131 (29.64%)	
HDL-C				0.041
Normal	707 (91.94%)	293 (89.60%)	414 (93.67%)	
Abnormal	62 (8.06%)	34 (10.40%)	28 (6.33%)	
LDL-C				0.261
Normal	417 (54.23%)	185 (56.57%)	232 (52.49%)	
Abnormal	352 (45.77%)	142 (43.43%)	210 (47.51%)	
BMI				0.642
Normal	350 (45.51%)	152 (46.48%)	198 (44.80%)	
Abnormal	419 (54.49%)	175 (53.52%)	244 (55.20%)	
Number of polyps at first detection, median [IQR]	2.000 [1.000, 4.000]	1.000 [1.000, 3.000]	2.000 [1.000, 4.000]	0.071
Volume of polyps at first detection (mm), median [IQR]	50.000 [10.800, 100.000]	25.600 [10.800, 100.000]	50.000 [10.800, 100.000]	0.096
Maximum diameter of polyps at first detection (mm), median [IQR]	8.000 [6.000, 12.000]	10.000 [6.000, 10.000]	8.000 [6.000, 12.000]	0.928
Surveillance interval (months), median [IQR]	13.000 [11.000, 17.000]	12.000 [10.000, 13.000]	14.000 [11.000, 18.000]	<0.001

Note: IQR, interquartile range; BMI, body mass index.

**Table 2:** Univariate and multivariate Cox regression analyses of factors associated with polyp recurrence in the training set.

Variable	Univariate Analysis		Multivariate Analysis	
	HR (95% CI)	*p* Value	HR (95% CI)	*p* Value
Age	1.002 (0.995–1.010)	0.564	NA	NA
Sex (male vs. female)	1.094 (0.929–1.290)	0.282	NA	NA
TC	1.025 (0.864–1.216)	0.779	NA	NA
TG	0.946 (0.797–1.122)	0.523	NA	NA
HDL-C	1.375 (0.997–1.895)	0.052	1.461 (1.059–2.017)	0.021
LDL-C	0.951 (0.814–1.111)	0.528	NA	NA
BMI	0.903 (0.773–1.056)	0.202	NA	NA
Number of baseline polyps	1.042 (1.017–1.067)	0.001	1.042 (1.017–1.068)	<0.001
Volume of baseline polyps	1.000 (1.000–1.001) †	<0.001	1.0005 (1.0003–1.0007)	<0.001
Maximum diameter of baseline polyps	1.021 (1.008–1.035)	0.002	NA	NA

Note: † The univariate HR for polyp volume is shown as 1.000 due to the small unit of measurement (mm^3^); the actual value is approximately 1.0005. NA indicates not included in the multivariate model; CI, confidence interval; HR, hazard ratio; BMI, body mass index.

**Table 3:** Discriminative performance of machine learning models in the training and test sets (95% CI).

Model	Training AUC (95% CI)	Training AUPRC (95% CI)	Test AUC (95% CI)	Test AUPRC (95% CI)
LR	0.714 (0.657–0.764)	0.937 (0.914–0.956)	0.751 (0.684–0.812)	0.928 (0.895–0.955)
ENET	0.719 (0.668–0.775)	0.939 (0.917–0.957)	0.747 (0.676–0.811)	0.927 (0.895–0.955)
RF	0.778 (0.719–0.839)	0.938 (0.912–0.962)	0.759 (0.679–0.835)	0.873 (0.820–0.921)
XGBoost	0.866 (0.827–0.900)	0.974 (0.963–0.983)	0.849 (0.783–0.910)	0.931 (0.902–0.956)
GBM	0.874 (0.840–0.906)	0.976 (0.967–0.984)	0.863 (0.798–0.922)	0.935 (0.906–0.958)
NN	0.500 (0.500–0.500)	0.853 (0.824–0.883)	0.500 (0.500–0.500)	0.791 (0.735–0.843)
SVM	0.686 (0.633–0.739)	0.935 (0.917–0.952)	0.665 (0.578–0.746)	0.868 (0.798–0.925)
Stacked Ensemble	0.733 (0.680–0.785)	0.940 (0.916–0.959)	0.766 (0.701–0.826)	0.936 (0.907–0.959)

Note: AUC, area under the receiver operating characteristic curve; AUPRC, area under the precision-recall curve; CI, confidence interval.

**Table 4:** Pairwise comparisons of AUCs among the top four models in the training set (DeLong test with Holm adjustment).

Comparison	Raw *p*-Value	Holm-Adjusted *p*-Value
XGBoost vs. GBM	0.4464	1
RF vs. GBM	0.0097	0.0486
RF vs. XGBoost	0.0314	0.1257
Stacked vs. GBM	0.8872	1
Stacked vs. XGBoost	0.5125	1
Stacked vs. RF	0.0016	0.0097

**Table 5:** Comparison of classification performance between XGBoost and GBM models based on optimal thresholds in the training set.

Model	Optimal Threshold	Sensitivity	Specificity	PPV	NPV	Accuracy	F1 Score
XGBoost	0.797	0.826	0.696	0.941	0.407	0.807	0.880
GBM	0.787	0.837	0.696	0.941	0.423	0.816	0.886

## Data Availability

The raw datasets used and/or analyzed during the current study are available from the corresponding authors on reasonable request.

## Abbreviations

The following abbreviations are used in this manuscript:

aOR	Adjusted odds ratio
AUC	Area under the receiver operating characteristic curve
AUPRC	Area under the precision-recall curve
BMI	Body mass index
CI	Confidence interval
CRC	Colorectal cancer
DCA	Decision curve analysis
ENET	Elastic Net
GBM	Gradient Boosting Machine
HDL-C	High-density lipoprotein cholesterol
LDL-C	Low-density lipoprotein cholesterol
LR	Logistic Regression
MAPK	Mitogen-activated protein kinase
NN	Neural Network
NPV	Negative predictive value
HR	Hazard ratio
PI3K/AKT	Phosphatidylinositol 3-kinase/protein kinase B
PPV	Positive predictive value
RF	Random Forest
SVM	Support Vector Machine
TC	Total cholesterol
TG	Triglycerides
VIF	Variance inflation factor
